# Cutaneous Coccidioidomycosis Mimicking Rosacea in Immunosuppressed Patient, Arizona, USA, 2024

**DOI:** 10.3201/eid3110.250010

**Published:** 2025-10

**Authors:** Sabine Obagi, Lauren E. Murphy, Aleksi J. Hendricks, Kim Hiatt, Allen Miraflor, Robert E. Cashman, Mohammad Fazel

**Affiliations:** University of Arizona College of Medicine, Tucson, Arizona, USA (S. Obagi, L.E. Murphy, A.J. Hendricks, M. Fazel); DermPath Diagnostics, Tucson (K. Hiatt, A. Miraflor, R.E. Cashman)

**Keywords:** Coccidioidomycosis, cutaneous coccidioidomycosis, fungi, fungal skin diseases, immunosuppression, histopathology, vandetanib, drug-induced hyperpigmentation, antifungal agents, fluconazole, endemic mycoses, United States

## Abstract

An immunocompromised patient in Arizona, USA, experienced cutaneous coccidioidomycosis mimicking rosacea-like dermatitis; she was receiving vandetanib therapy. Histopathology confirmed the diagnosis despite inconclusive serologic results. Lesions resolved with fluconazole treatment, but blue-gray hyperpigmentation persisted, likely linked to vandetanib. This case highlights diagnostic challenges in endemic fungal infections and rare drug-associated hyperpigmentation.

Coccidioidomycosis is caused by dustborne and soilborne *Coccidioides* spp. fungi that are endemic to the southwestern United States (*1*). *Coccidioides* spores become aerosolized and, when inhaled, develop into spherules within the lungs. The spherules can rupture and release endospores, enabling pulmonary spread and dissemination to other organs (*1*)*.*

Coccidioidomycosis manifests nonspecific symptoms: fever, cough, dyspnea, fatigue, arthralgias, myalgias, and cutaneous lesions. The differential diagnosis for cutaneous coccidioidomycosis includes rosacea, discoid lupus, psoriasis, sarcoidosis, granuloma faciale, and other disseminated fungal infections such as tuberculosis, paracoccidioidomycosis, and blastomycosis. We report cutaneous coccidioidomycosis mimicking rosacea-like dermatitis in an immunocompromised patient in Arizona, USA. 

A 70-year-old woman with stage IV medullary thyroid cancer being treated with the multikinase inhibitor vandetanib who had undergone thyroidectomy and radiation therapy was referred for an 8-month history of progressive pruritic, inflammatory papules and pustules on the central forehead and bilateral cheeks (Figure 1, panel A). The patient denied facial trauma. Before referral, she received a 10-day course of prednisolone, followed by empiric oral antibiotics (cephalexin, levofloxacin), antiviral drugs (acyclovir), and topical agents (clindamycin, clotrimazole), prescribed over 4 months by various providers, with transient or no improvement. Oral prednisolone treatment led to temporary improvement in the inflammatory lesions but subsequent worsening upon discontinuation. A review of systems revealed occasional diarrhea and night sweats. 

**Figure 1:**
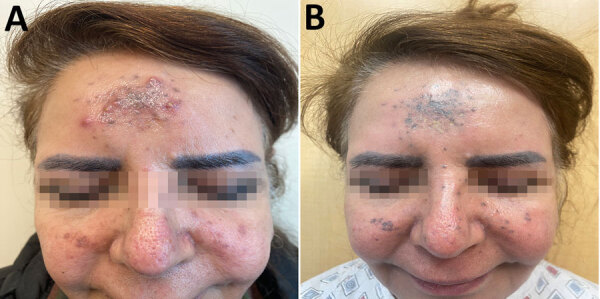
Clinical manifestations in case of cutaneous coccidioidomycosis mimicking rosacea in immunosuppressed patient, Arizona, USA, 2024. A) Inflammatory papules and pustules on the forehead and cheeks before treatment. B) Persistent blue-gray hyperpigmentation after 3 months of fluconazole therapy.

The patient, originally from Iran, had lived in Arizona for 5 years after previous residency in California and Virginia. At the time of her first dermatology visit (8 months after lesion onset), she was started on a 1-month course of doxycycline (100 mg 2×/d) and topical metronidazole (0.75% 2×/d) for a working diagnosis of rosacea, with no clinical improvement. 

Given the lack of response to standard rosacea therapies, at her second dermatology visit (≈9 months after initial symptoms), we performed a punch biopsy of a representative pustule on the left glabella. Histopathology demonstrated granulomatous inflammation with lymphocytes, neutrophils, and eosinophils, plus periodic acid–Schiff–positive spherules containing endospores (Figure 2), confirming a diagnosis of cutaneous coccidioidomycosis. Serologic testing yielded negative IgM and indeterminate IgG results. However, additional confirmatory tests (tissue culture and PCR) were deemed unnecessary given the patient’s residence in an endemic region and characteristic histopathologic findings. 

**Figure 2:**
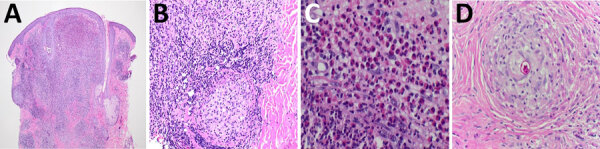
Histopathologic findings in case of cutaneous coccidioidomycosis mimicking rosacea in immunosuppressed patient, Arizona, USA, 2024. A–C) Granulomatous inflammation with mixed infiltrate of lymphocytes, histiocytes, neutrophils, and eosinophils. Hematoxylin and eosin stain; original magnifications ×2 (A), ×20 (B), and ×40 (C). D) Periodic acid–Schiff staining demonstrating a coccidioidal spherule containing endospores within a granuloma. Original magnification ×40.

Chest imaging revealed a new 1.3 × 1.3-cm pulmonary nodule within the left lower lobe among otherwise stable nodules. The nodules were subcentimeter and not biopsied. Given the patient’s oncologic history, it was unclear whether they represented metastatic disease or pulmonary coccidioidomycosis. Without definitive pathology, empiric antifungal therapy was initiated. After 2 weeks of fluconazole (400 mg/d), the papules and pustules showed substantial regression. However, after 3 months of oral fluconazole, blue-gray hyperpigmentation persisted at sites of prior papulopustules (Figure 1, panel B). After multidisciplinary discussion among infectious disease and oncology specialists, continuation of vandetanib and concurrent indefinite fluconazole treatment was recommended.

Given the absence of preceding trauma in this patient, direct inoculation with *Coccidioides* spp. is unlikely. Hematogenous dissemination is favored, particularly considering the patient’s immunosuppression from both malignancy and tyrosine kinase inhibitor therapy. Her recent relocation to Arizona without early-life endemic exposure might have limited immune priming and increased susceptibility.

We histopathologically identified coccidioidomycosis in this case. Coccidioidomycosis shows refractile spherules with endospores, whereas paracoccidioidomycosis shows pilot wheel budding and blastomycosis shows broad-based budding. Histopathology may show granulomatous dermatitis, eosinophilic inflammation, micro-abscesses, perivascular inflammation, and gummatous necrosis. Spherules are typically identifiable by using methenamine silver, periodic acid–Schiff stains, or Papanicolaou stains (*2*)*.* Immunosuppression increases risk for disseminated coccidioidomycosis; only 1% of immunocompetent persons have disseminated disease develop, compared with 30%–50% of immunosuppressed persons (*2*)*.* Patients who are immunocompromised (e.g., by HIV, malignancy, organ transplantation, antiinflammatory biologics, or chemotherapy) face 50% mortality with disseminated disease (*3*)*.* Coccidioidomycosis can relapse during immunosuppressive treatment after antifungal discontinuation (*4*)*.* Treatment varies by host immunity and organ involvement, ranging from observation to amphotericin B (*5*). Secondary cutaneous involvement occurs in up to 15% of extrapulmonary cases, especially in immunocompromised patients (*6*). Lesions mimic other dermatologic conditions, requiring histologic confirmation. 

Azoles represent first-line therapy for cutaneous coccidioidomycosis; response rates are 25%–91% (*5*)*.* This patient improved substantially with fluconazole at a dose of 400 mg/day. Immunocompetent patients typically need 6–12 months of therapy, whereas immunocompromised patients with disseminated disease often require lifelong treatment, depending on severity and immunosuppression (*5*)*.* Given variable responses and relapse risk, careful monitoring is essential after treatment discontinuation. 

This patient had persistent blue-gray hyperpigmentation develop at sites of prior papulopustules, intensifying after 3 months of fluconazole (Figure 1, panel B). Tetracyclines, especially minocycline, can cause hyperpigmentation, and blue-gray hyperpigmentation has been reported with vandetanib. Evidence suggests potential synergistic hyperpigmentation with concurrent vandetanib and doxycycline use (*7*)*.*

In summary, this case highlights diagnostic challenges in endemic fungal infections and rare drug-associated hyperpigmentation. Diagnostic stewardship, particularly in endemic regions, is critical to reduce delays in appropriate antifungal therapy and minimize unnecessary antimicrobial exposure.

## References

[1] Centers for Disease Control and Prevention. Where valley fever (coccidioidomycosis) comes from. August 10, 2021 [cited 2023 Feb 25]. https://www.cdc.gov/fungal/diseases/coccidioidomycosis/causes.html

[2] Quimby SR, Connolly SM, Winkelmann RK, Smilack JD. Clinicopathologic spectrum of specific cutaneous lesions of disseminated coccidioidomycosis. J Am Acad Dermatol. 1992;26:79–85. 10.1016/0190-9622(92)70011-4 1732341

[3] Odio CD, Marciano BE, Galgiani JN, Holland SM. Risk factors for disseminated coccidioidomycosis, United States. Emerg Infect Dis. 2017;23:308–11. 10.3201/eid2302.160505 28098554 PMC5324825

[4] Blair JE, Ampel NM, Hoover SE. Coccidioidomycosis in selected immunosuppressed hosts. Med Mycol. 2019;57(Supplement_1):S56–63. 10.1093/mmy/myy019 29669037

[5] Galgiani JN, Ampel NM, Blair JE, Catanzaro A, Geertsma F, Hoover SE, et al. 2016 Infectious Diseases Society of America (IDSA) clinical practice guideline for the treatment of coccidioidomycosis. Clin Infect Dis. 2016;63:e112–46. 10.1093/cid/ciw360 27470238

[6] Galgiani JN, Ampel NM, Blair JE, Catanzaro A, Johnson RH, Stevens DA, et al.; Infectious Diseases Society of America. Coccidioidomycosis. Clin Infect Dis. 2005;41:1217–23. 10.1086/496991 16206093

[7] Perlmutter JW, Cogan RC, Wiseman MC. Blue-grey hyperpigmentation in acne after vandetanib therapy and doxycycline use: a case report. SAGE Open Med Case Rep. 2022;10:2050313X221086316. 10.1177/2050313X221086316 PMC8943637 35341100

